# Fungal Pretreatment-Assisted Methane Recovery from Food Waste: A Literature-Informed Simulation Framework

**DOI:** 10.3390/molecules31203594

**Published:** 2026-10-09

**Authors:** Pouya Zaferanloo, Mohammad Imamul Hossain, Shoji Maehara, Mehran Ektesabi, François Malherbe, Bita Zaferanloo

**Affiliations:** 1 School of Engineering, Department of Engineering Technologies, Swinburne University of Technology, Melbourne, VIC 3122, Australia; 2 Graduate Researcher, School of Engineering, Faculty of Science, Engineering and Built Environment, Deakin University, Burwood, VIC 3125, Australia; 3 Faculty of Pharmacy and Pharmaceutical Sciences, Fukuyama University, Fukuyama, Hiroshima 729-0292, Japan; 4 School of Science, Computing and Emerging Technologies, Department of Chemistry and Biotechnology, Swinburne University of Technology, Melbourne, VIC 3122, Australia

**Keywords:** food waste, fungal pretreatment, methane recovery, anaerobic digestion, spent coffee grounds, fungal enzymes, enzyme-mediated hydrolysis, literature-informed modelling, scenario screening, circular bioeconomy

## Abstract

Food waste is a heterogeneous, moisture-rich substrate with substantial potential for renewable methane recovery, but variability in composition, hydrolysis and reactor stability constrains reliable anaerobic-digestion performance. This study presents a literature-informed computational scenario-screening framework that translates fungal pretreatment descriptors into model-ready variables for anaerobic digestion. Published experimental and modelling studies were used to parameterise food-waste composition, spent coffee grounds (SCG), enzyme-targeted pretreatment, anaerobic-digestion operating conditions, methane potential and heuristic risk indicators; no new experimental measurements were generated. The Python-based framework links fungal pretreatment severity with substrate solubilisation, hydrolysis, methane yield, raw-biogas CH_4_ fraction, gross methane energy recovery and operational-risk indicators. Under the specified model assumptions, heuristic stability-adjusted methane output methane screening output increased from 272.5 L CH_4_ kg^−1^ VS in the untreated control to 291.5 L CH_4_ kg^−1^ VS under high pretreatment and 295.5 L CH_4_ kg^−1^ VS under extended pretreatment, while increasing SCG inclusion and high organic loading reduced the corresponding screening outputs. These directional responses are partly imposed by the model structure and are therefore interpreted as comparative scenario outputs and experimentally testable hypotheses rather than independent quantitative predictions or process optima.

## 1. Introduction

### 1.1. Food Waste, Climate Impact, and Renewable-Energy Opportunity

Food waste is both an environmental liability and a lost carbon and nutrient resource. The United Nations Environment Programme estimated that approximately 1.05 billion tonnes of food were wasted at household, food service and retail levels in 2022, while food loss and waste together account for roughly 8–10% of global greenhouse-gas emissions [1]. Because food waste is generally moisture-rich and biodegradable, anaerobic digestion (AD) is a particularly relevant recovery route: it avoids the drying demand of many thermochemical processes and produces methane-rich biogas that can be used for heat and power or upgraded to biomethane [2,3,4]. In this context, AD should complement, rather than replace, prevention and redistribution strategies, with its strongest role being the valorisation of unavoidable or non-edible residual streams [1,4].

The renewable-energy opportunity therefore extends beyond waste disposal. Food waste can be redirected into a controlled biochemical conversion pathway in which feedstock composition, pretreatment and operating conditions determine methane recovery and process stability. Mathematical modelling can support this transition by connecting substrate chemistry with conversion kinetics, gas production, energy recovery and operational risk [5,6].

### 1.2. Anaerobic Digestion and Methane Recovery

AD is conventionally represented as a sequence of disintegration/hydrolysis, acidogenesis, acetogenesis and methanogenesis, with gas–liquid transfer and physicochemical equilibria influencing final gas composition. The Anaerobic Digestion Model No. 1 (ADM1) remains a benchmark mechanistic framework and explicitly partitions particulate organic matter into carbohydrate-, protein- and lipid-related fractions that are converted to soluble intermediates and, ultimately, methane and carbon dioxide [7]. In the present study, ADM1 is used as a mechanistic reference point rather than as the implemented model. The fungal pretreatment severity index (FPSI) does not replace ADM1; instead, it acts as an upstream heuristic translation layer that converts pretreatment descriptors into changes in substrate accessibility, solubilisation, hydrolysis and biodegradability for scenario screening. Food-waste methane yield depends on substrate composition, accessibility, inoculum quality and operating conditions, while raw biogas commonly contains approximately 50–70% CH_4_ before upgrading [2,8].

For simulation studies, feedstock characterisation is critical. Reviews of AD modelling emphasise that credible prediction requires appropriate substrate fractionation, calibration strategy and representation of inhibition and alternative pathways [5,9]. These needs are central to the present study because fungal pretreatment is treated as a biochemical modification of the substrate rather than as an isolated upstream step.

### 1.3. Limitations of Direct Digestion of Complex Food Waste

Food waste is compositionally heterogeneous, with substantial variation in total solids (TS), volatile solids (VS), chemical oxygen demand (COD), soluble COD (SCOD), particle size, carbohydrate, protein, lipid and fibre fractions. Source and geography can materially change its physicochemical and biochemical characteristics [10,11]. Rapid fermentation of readily degradable carbon can lead to volatile fatty acid (VFA) accumulation and pH decline, while protein-rich streams can increase ammonia-related inhibition and lipid-rich streams can generate long-chain fatty acids that inhibit syntrophic and methanogenic activity at elevated loading [12,13]. In the present framework, these mechanisms are represented only through simplified heuristic risk terms; they are not full inhibition kinetics and should not be interpreted as experimentally validated predictions of reactor stability. These constraints explain the continuing interest in pretreatment, co-digestion and loading-rate control.

### 1.4. Spent Coffee Grounds as a Co-Substrate and Fungal-Pretreatment Target

Spent coffee grounds (SCG) are a useful model co-substrate because they combine valorisation potential with structural recalcitrance. SCG retains substantial polysaccharides, lipids, proteins and bioactive compounds after brewing, including phenolics and caffeine that can complicate direct anaerobic conversion [14,15,16]. Recent characterisation confirms that SCG can contain substantial cellulose, hemicellulose and lignin fractions, while coffee oil remains a significant lipid fraction [17,18].

SCG also provides a practical stress test for fungal pretreatment because hydrolytic and oxidative fungal enzymes can target structural polysaccharides, lignin-like barriers and associated phenolic material. Importantly, continuous co-digestion research found that small SCG additions can increase methane production, whereas increasing the SCG fraction can produce diminishing returns or instability depending on process conditions [19]. Accordingly, the 10–30% SCG scenarios used here are treated as screening and stress cases, not as literature-defined optimum inclusion rates.

### 1.5. Study Aim, Novelty, and Scope

The present study develops and evaluates a literature-informed computational framework that connects fungal-biotechnology knowledge with AD process simulation. Its novelty lies not in proposing a new validated AD kinetic model, but in providing an explicit and reproducible translation of fungal enzyme-related activity, pretreatment time, temperature and pH into model-ready descriptors of solubilisation, hydrolysis, lag-phase modification, biodegradability, methane production, raw-biogas CH_4_ fraction, gross methane energy recovery and operational risk. This creates a transparent sequential pathway from fungal pretreatment assumptions to substrate modification and then to downstream AD screening outputs, rather than linking enzyme dose directly to methane yield.

All biochemical and engineering inputs were obtained from published experimental or modelling literature; no new laboratory measurements were generated. The framework is evaluated through literature-based consistency checks and deterministic scenario analysis, not independent experimental calibration or validation. The current study therefore aims to identify internally consistent candidate scenarios and experimentally testable hypotheses for a subsequent laboratory phase rather than to provide quantitatively validated operating predictions.

For clarity, the framework is used throughout as a literature-informed scenario-screening tool rather than a calibrated reactor model. The Results present outputs from the final S1–S9 scenario run, while the full equations, parameter definitions and modelling assumptions are provided in Section 4. Abbreviations are defined at first use and retained in the consolidated abbreviation list at the end of the manuscript.

## 2. Results

### 2.1. Literature-Derived Biochemical and Fungal-Biotechnology Basis for Model Development

#### 2.1.1. Organic-Matter Composition and Methane Potential

Food waste can be represented by major biodegradable organic fractions—carbohydrates, proteins and lipids—together with an inert fraction. Their relative proportions vary widely among waste streams [10,11]. This fractionation is useful for modelling because each biochemical class differs in hydrolysis behaviour, stoichiometric methane potential and inhibition risk [7,8]. SCOD is used as a proxy for the soluble, oxidisable fraction of organic matter and can therefore indicate increased substrate accessibility after pretreatment; it should not be interpreted as a direct consequence of the elemental oxygen content of the substrate.

#### 2.1.2. Polysaccharide-Rich Fractions

Starch, cellulose, hemicellulose and pectin are common carbohydrate components of plant-derived food residues. Starch is comparatively accessible to amylolytic enzymes, which cleave α-glycosidic linkages and release shorter oligosaccharides and fermentable sugars. Cellulose requires coordinated action of endoglucanases, exoglucanases/cellobiohydrolases and β-glucosidases, whereas hemicellulose degradation depends on xylanases, mannanases and accessory debranching enzymes. In lignocellulosic matrices, oxidative and lignin-modifying fungal systems can further increase enzyme access to structural polysaccharides [20,21]. From a modelling perspective, these mechanisms provide the biological rationale for linking fungal pretreatment to increased SCOD, soluble-sugar release and hydrolysis-related response terms.

#### 2.1.3. Lipid-Rich Fractions

Lipids have a high theoretical methane potential because of their reduced carbon state, but their hydrolysis can generate long-chain fatty acids that inhibit microbial conversion at excessive loading [8,13]. Lipolytic fungi and yeasts such as *Yarrowia lipolytica* secrete lipases and can metabolise hydrophobic substrates, while *Starmerella bombicola* is an industrially relevant lipid-metabolising yeast [22,23]. These organisms are cited only as biological examples demonstrating the plausibility of lipid-targeting fungal/yeast enzyme systems; neither organism is represented explicitly in the simulation, and no species-specific growth or enzyme-secretion kinetics are used. In the present framework, lipid-targeting activity is represented generically within the pretreatment severity layer, while downstream risk terms account heuristically for potential process stress.

#### 2.1.4. Protein-Rich Fractions

Proteinaceous food-waste fractions are hydrolysed to peptides and amino acids, supplying soluble carbon and nitrogen but potentially increasing ammonia-related inhibition after further conversion. Aspergillus oryzae is a well-established industrial protease producer, and solid-state fermentation can generate high protease activities on agro-industrial substrates [24]. It is cited here as an example supporting the biological plausibility of protease-mediated pretreatment rather than as an organism explicitly simulated in the framework. The model does not include species-specific fungal growth or protease-secretion kinetics. It instead distinguishes soluble nitrogen released during pretreatment (free amino nitrogen) from total ammonia nitrogen (TAN) and free ammonia, which are relevant to AD inhibition.

#### 2.1.5. Fungal Pretreatment and Enzyme-Mediated Conversion

**Figure 1:**
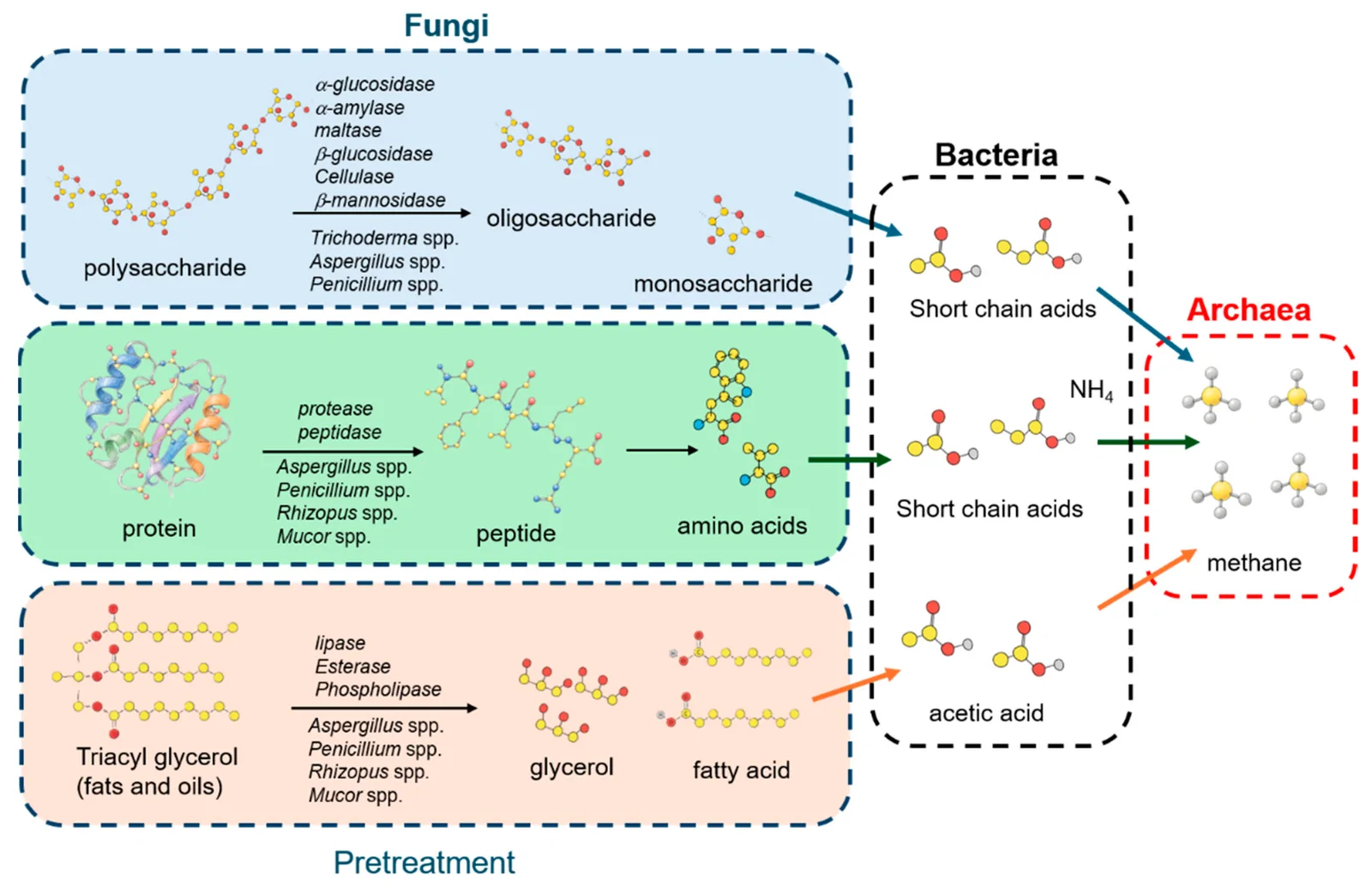
Conceptual pathway linking fungal pretreatment of polysaccharide-, protein-, and lipid-rich food-waste fractions with downstream bacterial conversion and archaeal methanogenesis. The blue, green, and orange shaded regions represent polysaccharide-, protein-, and lipid-related fungal pretreatment pathways, respectively. The black dashed box represents downstream bacterial conversion, while the red dashed box represents archaeal methanogenesis. Coloured arrows indicate the transfer of products derived from the corresponding substrate pathways into downstream bacterial and archaeal conversion steps, while arrows within each region indicate the direction of biochemical conversion. The diagram represents the model architecture and is not intended as a complete microbial-network model.

Fungal pretreatment is attractive because filamentous fungi can penetrate solid biomass while secreting extracellular hydrolytic and oxidative enzymes. Lignocellulose-degrading fungi deploy cellulases, hemicellulases, lytic polysaccharide monooxygenases and lignin-modifying systems that can increase substrate accessibility [20]. White-rot fungi add oxidative enzymes such as laccase, lignin peroxidase, manganese peroxidase and versatile peroxidase, which can modify lignin and phenolic structures [25,26]. These mechanisms provide the biochemical rationale for representing fungal treatment as an upstream severity layer that modifies substrate accessibility rather than modelling fungi as a methane-producing population. FPSI has not been calibrated against fungal biomass dynamics, measured enzyme-secretion kinetics or species-specific population models; the relationship between pretreatment conditions and fungal enzyme production is therefore represented implicitly through empirical response functions. FPSI should accordingly be interpreted as a heuristic composite screening index. Collectively, these fungal enzyme-mediated transformations provide the upstream biochemical link between complex food-waste fractions and the soluble intermediates subsequently processed by bacterial communities and methanogenic archaea, as conceptualised in Figure 1.

#### 2.1.6. Fungal Pretreatment of Spent Coffee Grounds

**Figure 2:**
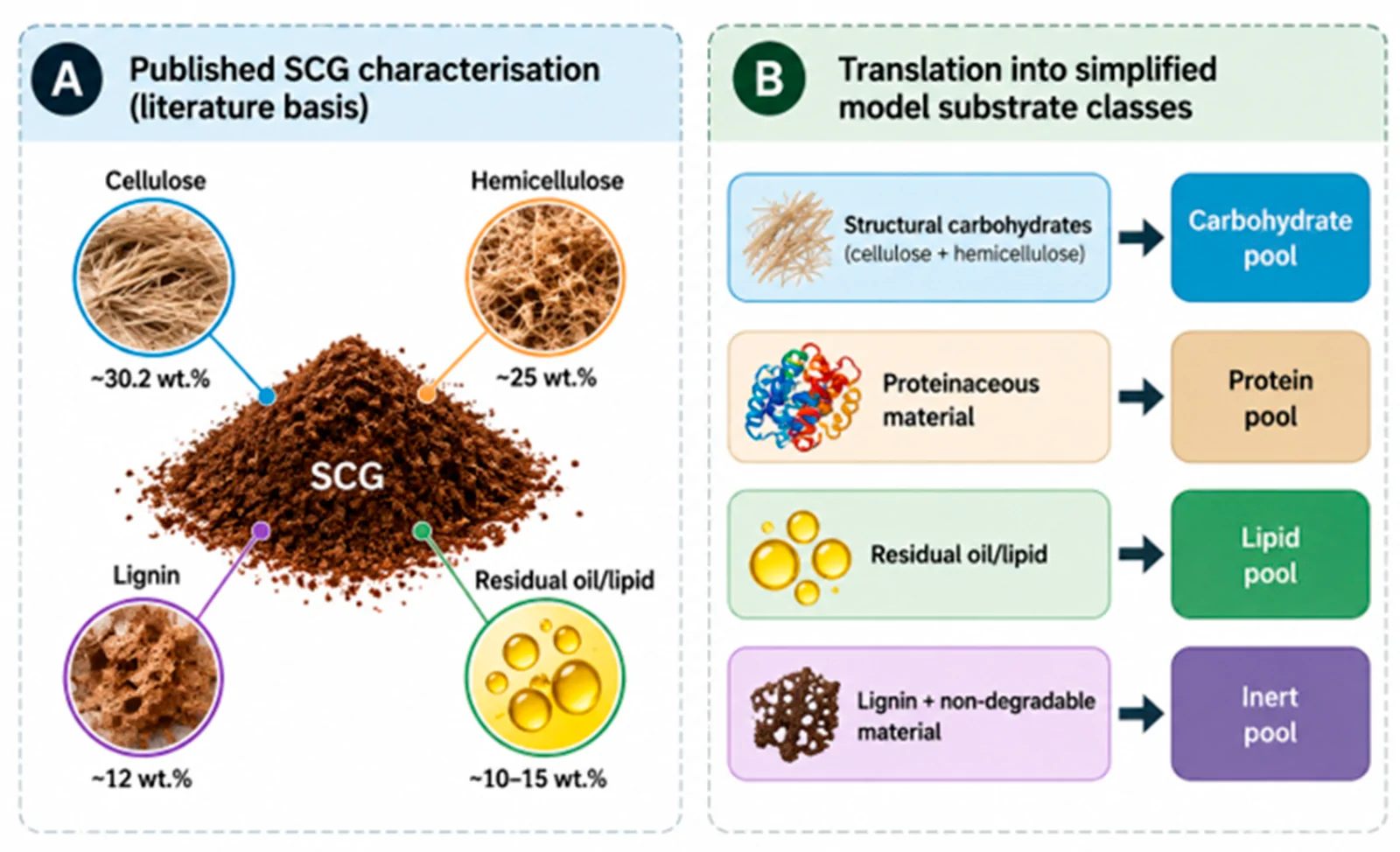
Literature-informed representation of spent coffee grounds (SCG) composition and its translation into simplified model substrate classes. (**A**) Published SCG characterisation reported approximately 30.2 wt.% cellulose, 25 wt.% hemicellulose and 12 wt.% lignin [17], while residual oil contents vary with coffee type and processing conditions [18]. These values originate from different analytical datasets and are not intended to represent a complete composition summing to 100%. (**B**) Conceptual translation of literature-reported SCG constituents into the simplified carbohydrate, protein, lipid and inert substrate classes used in the simulation. The corresponding model fractions and their literature basis or modelling-assumption status are reported separately in Table 1.

SCG is chemically suited to this framework because it contains both fermentable and recalcitrant constituents. Published structural characterisation reported approximately 30.2 wt.% cellulose, 25 wt.% hemicellulose and 12 wt.% lignin in an SCG sample set [17], while coffee-oil studies report residual lipid contents that vary with coffee type and processing conditions [18]. These literature values describe source-specific structural composition and are not directly equivalent to the simplified carbohydrate/protein/lipid/inert fractions used by the simulation. Figure 2 therefore separates literature-reported SCG characterisation from its conceptual translation into the model substrate classes. White-rot fungal pretreatment has been shown to increase accessibility of lignocellulosic biomass and enhance subsequent biological conversion, supporting the use of oxidative enzymes as SCG-supporting pretreatment variables [27].

**Table 1:** Fixed model parameters, coefficients and assumption status used in the final literature-informed screening simulation.

Parameter	Value	Unit	Role in Model	Source/Status
Food-waste composition	0.50 carb; 0.25 protein; 0.20 lipid; 0.05 inert	fraction of VS	Baseline substrate partition	Selected within literature-reported ranges [2,10,13]; literature-informed baseline
Food-waste baseline biodegradability	0.72	fraction	Untreated biodegradability prior	Author-selected literature-informed screening prior; requires calibration
SCG model composition	0.42 carb; 0.16 protein; 0.22 lipid; 0.20 inert	fraction of VS	Simplified SCG substrate partition	Literature-informed modelling assumption bounded by SCG literature [14,15,16,17,18,19]; not a direct reproduction of Figure 2A
SCG baseline biodegradability	0.55	fraction	Untreated SCG biodegradability prior	Author-selected screening prior; requires calibration
Baseline OLR/critical OLR	2.5/5.0	kg VS m^−3^ d^−1^	Operating input/risk normalisation	Baseline selected within literature range [28,29,30]; critical value is an internal screening threshold
HRT	25 (S1–S8); 20 (S9)	d	Scenario descriptor	Literature-informed range [28,29]; no direct equation effect in current model
AD temperature/initial pH/pH optimum	35/7.2/7.1	°C/-/-	Baseline digestion conditions and pH risk	Literature-informed baseline [28,29]
Control SCOD/sugar/FAN/TAN/VFA	18/4/180/1200/1800	g L^−1^/g L^−1^/mg L^−1^/mg L^−1^/mg L^−1^	Reference chemistry priors	Internal literature-informed screening priors; not experimentally calibrated
Critical VFA/FAN/TAN	6000/600/3500	mg L^−1^	Risk normalisation thresholds	Internal literature-informed screening thresholds informed by inhibition literature [12,13,31]
Carbohydrate fungal response	K_E = 50; T_opt = 30; σ_T = 8; pH_opt = 5.5; σ_pH = 1.0; k_t = 0.050; w = 0.35	U g^−1^ VS; °C; °C; -; -; h^−1^; -	FPSI carbohydrate response	Author-selected heuristic values; biologically motivated by [16,20,32,33,34]; requires calibration
Protein fungal response	K_E = 45; T_opt = 32; σ_T = 8; pH_opt = 6.0; σ_pH = 1.0; k_t = 0.040; w = 0.25	U g^−1^ VS; °C; °C; -; -; h^−1^; -	FPSI protein response	Author-selected heuristic values; requires calibration
Lipid fungal response	K_E = 60; T_opt = 30; σ_T = 8; pH_opt = 5.5; σ_pH = 1.2; k_t = 0.030; w = 0.20	U g^−1^ VS; °C; °C; -; -; h^−1^; -	FPSI lipid response	Author-selected heuristic values; requires calibration
SCG-supporting fungal response	K_E = 70; T_opt = 28; σ_T = 10; pH_opt = 5.0; σ_pH = 1.2; k_t = 0.035; w = 0.20	U g^−1^ VS; °C; °C; -; -; h^−1^; -	FPSI SCG response	Author-selected heuristic values; requires calibration
Enzyme allocation factors	0.40 carb; 0.30 protein; 0.20 lipid; 0.10 SCG	fraction	Distributes total nominal enzyme activity	Internal allocation assumption; requires enzyme-specific measurements
Solubilisation coefficients	α_SCOD = 0.80; α_sugar = 1.20; α_FAN = 0.70; α_TAN = 0.20	dimensionless	Pretreatment-to-solubilisation response	Author-selected heuristic coefficients; require calibration
Hydrolysis/lag coefficients	α_hyd = 1.50; α_λ = 0.55	dimensionless	FPSI modification of hydrolysis and lag	Author-selected heuristic coefficients; require calibration
VFA and pH response coefficients	0.55 FPSI; 0.25 OLR; 0.45 pH	dimensionless	Heuristic VFA and pH estimation	Internal screening coefficients informed by inhibition behaviour [12,13,31]; not fitted
Stability risk weights	0.35 VFA; 0.25 nitrogen; 0.20 pH; 0.20 OLR	dimensionless	Weighted risk score	Author-selected heuristic weights; not calibrated
Raw-biogas CH_4_ equation	0.58 + 0.08 FPSI − 0.05 R_total − 0.03 f_SCG; clipped 0.50–0.70	fraction	Raw-biogas methane fraction	Heuristic screening expression bounded by typical food-waste biogas range [2,8,29]
CHP electrical efficiency	0.35	fraction	Electricity potential estimate	Assumed engineering efficiency; used for gross-energy screening only
Objective risk penalty, λ	0.80	dimensionless	Comparative objective score	Author-selected heuristic penalty; comparative ranking only

#### 2.1.7. Model-Ready Biological Variables

The fungal-biotechnology layer is represented by enzyme-targeted activity, pretreatment time, pretreatment temperature and pH; the conceptual pathway from fungal substrate modification to downstream methanogenesis is summarised in Figure 1. The selected variables are supported by studies showing that fungal or enzyme-rich pretreatment can increase glucose, free amino nitrogen and SCOD while improving downstream methane production [32]. Broader fungal pretreatment literature also indicates that treatment intensity and duration must be controlled because biological pretreatment can introduce time, contamination and substrate-loss penalties [16]. These considerations inform the fungal pretreatment severity index (FPSI) described in Section 4.4.

### 2.2. Simulation Framework

#### 2.2.1. Scenario Overview

One final simulation run was performed in Python 3.13.15 using NumPy 2.1.3, pandas 2.2.3, and Matplotlib 3.10.0, and was used as the master S1–S9 dataset. The scenarios compare untreated food waste, increasing combined fungal pretreatment severity, extended pretreatment, SCG co-substrate inclusion and a high-OLR operating stress case. Table 2 summarises the principal outputs used throughout the manuscript; the exact scenario inputs and the equations underlying FPSI, methane, risk and sensitivity calculations are provided in Section 4.3, Section 4.4, Section 4.5, Section 4.6, Section 4.7 and Section 4.8. This separation follows the journal structure while allowing the Results to be interpreted against explicitly defined model assumptions.

**Table 2:** Selected S1–S9 scenario definitions, inputs and deterministic screening outputs. (**A**) Scenario inputs. (**B**) Principal model outputs. Manuscript values are rounded to a precision appropriate for scenario screening; exact machine-precision outputs are archived in Zenodo [35].

(**A**) Scenario inputs. Pretreated scenarios use a pretreatment temperature of 30 °C and pH 5.5. HRT is retained as a scenario descriptor but does not enter the current methane or stability equations; therefore, the S9 model difference relative to S6 is generated by the higher OLR term rather than by HRT.										
**Scenario**	**Definition**				**Enzyme Dose/Time** **(U g^−1^ VS/h)**		**SCG**	**OLR** **(kg VS m^−3^ d^−1^)**		**HRT** **(d)**
S1	Control food waste; no pretreatment				0/0		0	2.5		25
S2	Low fungal pretreatment				30/12		0	2.5		25
S3	Moderate fungal pretreatment				60/24		0	2.5		25
S4	High fungal pretreatment				90/36		0	2.5		25
S5	Extended fungal pretreatment				90/72		0	2.5		25
S6	Moderate pretreatment + 10% SCG				60/24		10	2.5		25
S7	Moderate pretreatment + 20% SCG				60/24		20	2.5		25
S8	Moderate pretreatment + 30% SCG				60/24		30	2.5		25
S9	Moderate pretreatment + 10% SCG + high OLR				60/24		10	4.5		20
(**B**) Principal model outputs.										
**Scenario**		**FPSI**	**Unpenalised Biochemical Methane** **(L CH_4_ kg^−1^ VS)**	**Stability-Adjusted Methane Screening Output** **(L CH_4_ kg^−1^ VS)**		**Gross Energy** **(kWh; 2.25 kg VS)**			**Heuristic Stability**	
S1		0.000	384.9	272.48		6.1			0.71	
S2		0.048	392.8	276.89		6.2			0.71	
S3		0.129	407.4	284.38		6.4			0.70	
S4		0.205	421.2	291.46		6.5			0.69	
S5		0.248	428.9	295.54		6.6			0.69	
S6		0.131	395.2	275.87		6.2			0.70	
S7		0.134	383.2	267.47		6.0			0.70	
S8		0.136	371.3	259.19		5.8			0.70	
S9		0.131	395.4	235.67		5.3			0.60	

#### 2.2.2. Heuristic Stability-Adjusted Methane Screening Response to Fungal Pretreatment and SCG

**Figure 3:**
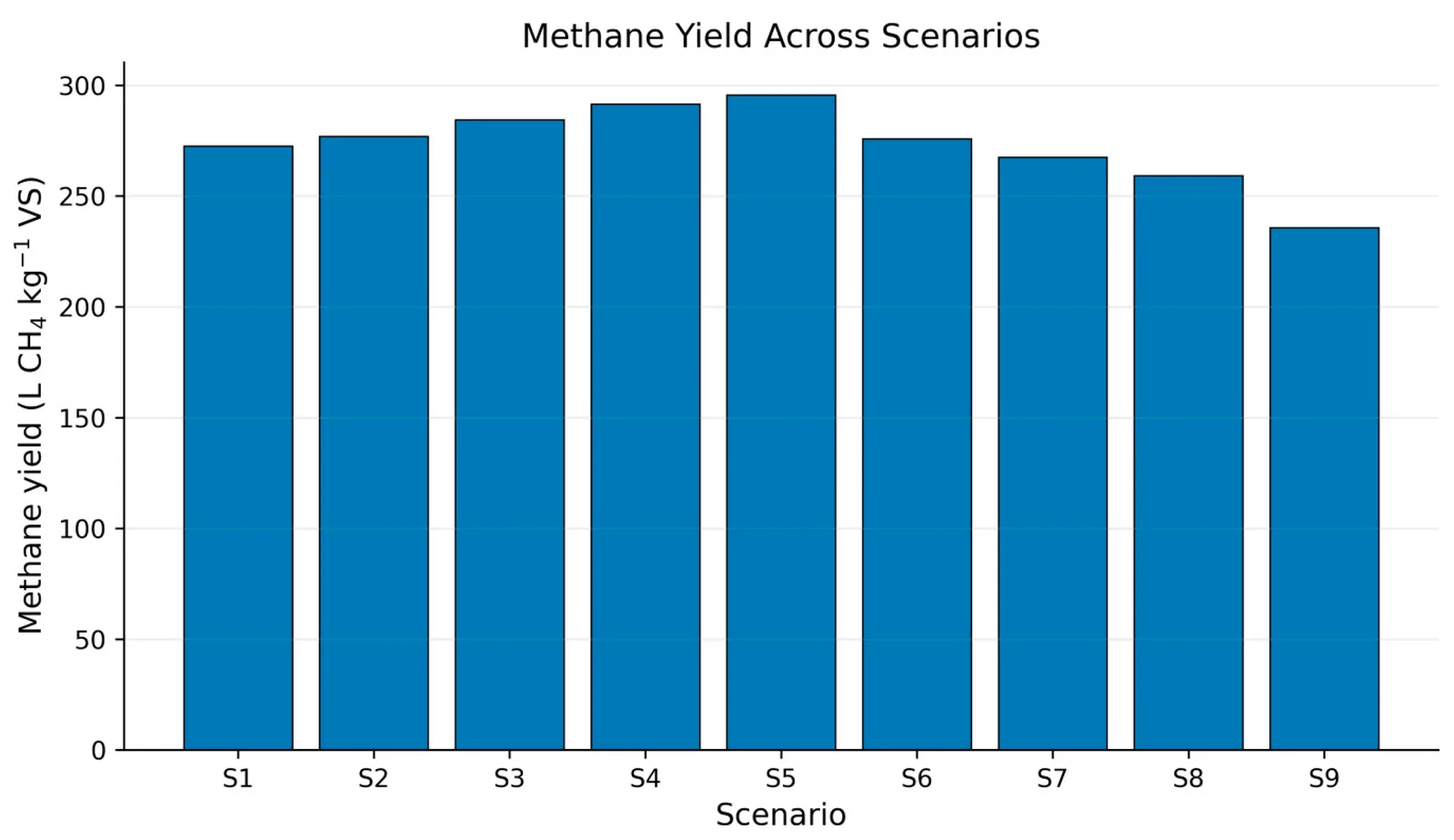
Modelled stability-adjusted methane output across the final S1–S9 literature-informed scenarios. S1, untreated control; S2–S5, low, moderate, high and extended fungal pretreatment; S6–S8, moderate pretreatment with 10%, 20% and 30% SCG; S9, moderate pretreatment with 10% SCG under high OLR. The values are deterministic scenario outputs rather than experimentally validated predictions.

Under the specified model relationships, stability-adjusted methane output increased across S2-S5, from 272.5 L CH_4_ kg^−1^ VS in S1 to 284.4 L CH_4_ kg^−1^ VS under moderate pretreatment, 291.5 L CH_4_ kg^−1^ VS under high pretreatment and 295.5 L CH_4_ kg^−1^ VS under extended pretreatment (Figure 3). These changes are deterministic consequences of the specified FPSI-to-solubilisation, biodegradability and risk relationships and are not independent evidence of a causal pretreatment effect. In the controlled SCG screening series S6–S8, moderate pretreatment and OLR were held constant while SCG inclusion increased from 10% to 30%; stability-adjusted methane output decreased from 275.9 to 259.2 L CH_4_ kg^−1^ VS. FPSI remained similar across S6–S8 because it represents pretreatment severity rather than SCG proportion itself. The change in methane output therefore arises through the mixed-substrate composition, assigned biodegradability, component methane potential and downstream risk terms. The high-OLR stress scenario generated the lowest stability-adjusted output, 235.7 L CH_4_ kg^−1^ VS.

#### 2.2.3. Cumulative Methane Production and Lag-Phase Behaviour

**Figure 4:**
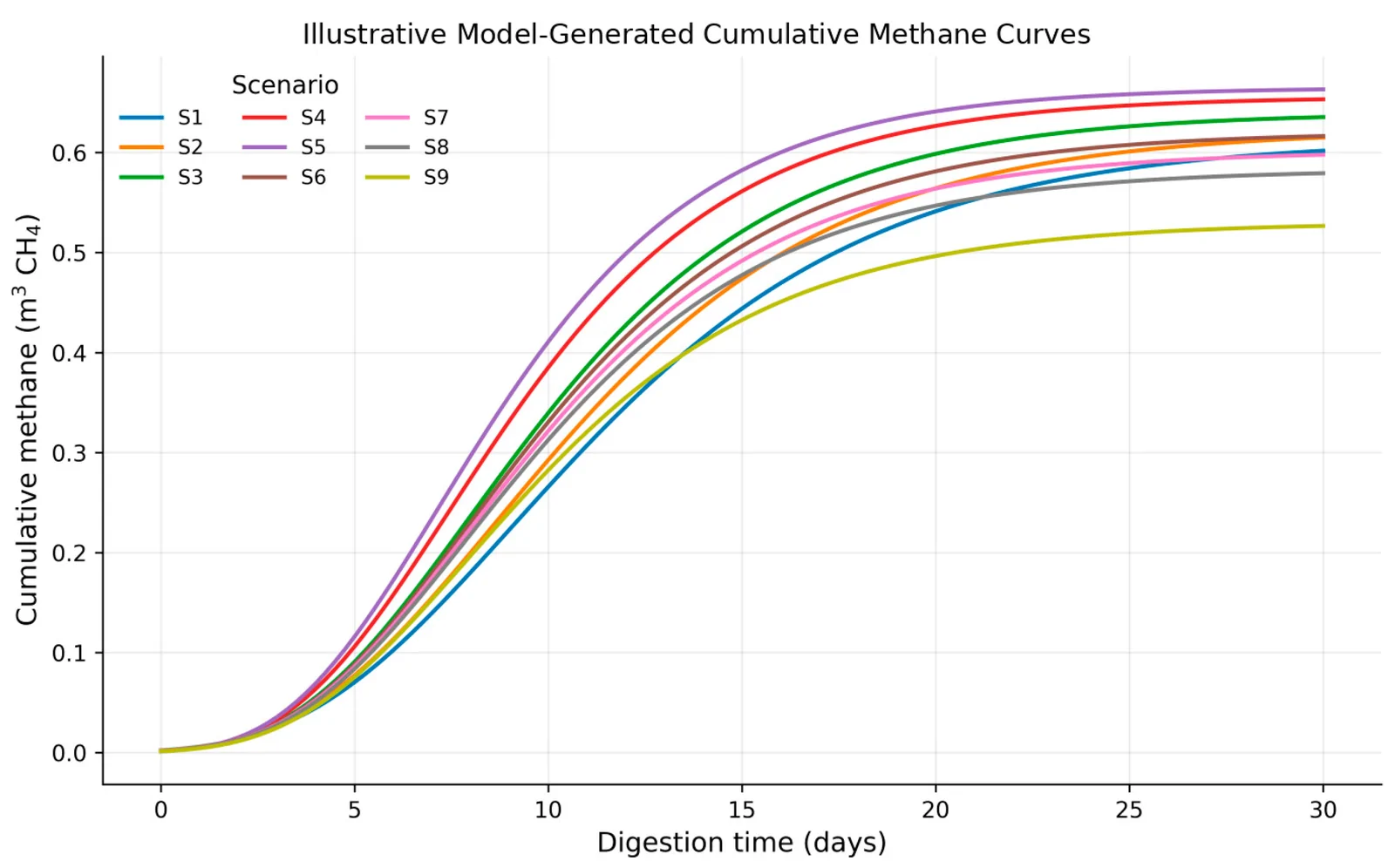
Illustrative model-generated cumulative methane trajectories over 30 days using the modified Gompertz formulation [36]. Curves use the stability-adjusted methane potential and FPSI-linked lag parameters and were not fitted to experimental BMP data; faster rise at higher FPSI is partly imposed by the model equations.

The modified Gompertz representation generated earlier methane accumulation as fungal pretreatment severity increased (Figure 4). High and extended pretreatment scenarios reached higher modelled cumulative methane volumes than the untreated control, whereas the high-OLR condition showed lower stability-adjusted methane accumulation. These curves are illustrative trajectories derived from the model outputs and were not fitted to experimental BMP measurements. Importantly, the lag parameter is explicitly modified as a function of FPSI; consequently, the shorter lag at higher FPSI is a structural consequence of the assumed framework rather than an independently observed kinetic effect.

#### 2.2.4. Modelled CH_4_ Fraction in Raw Biogas

**Figure 5:**
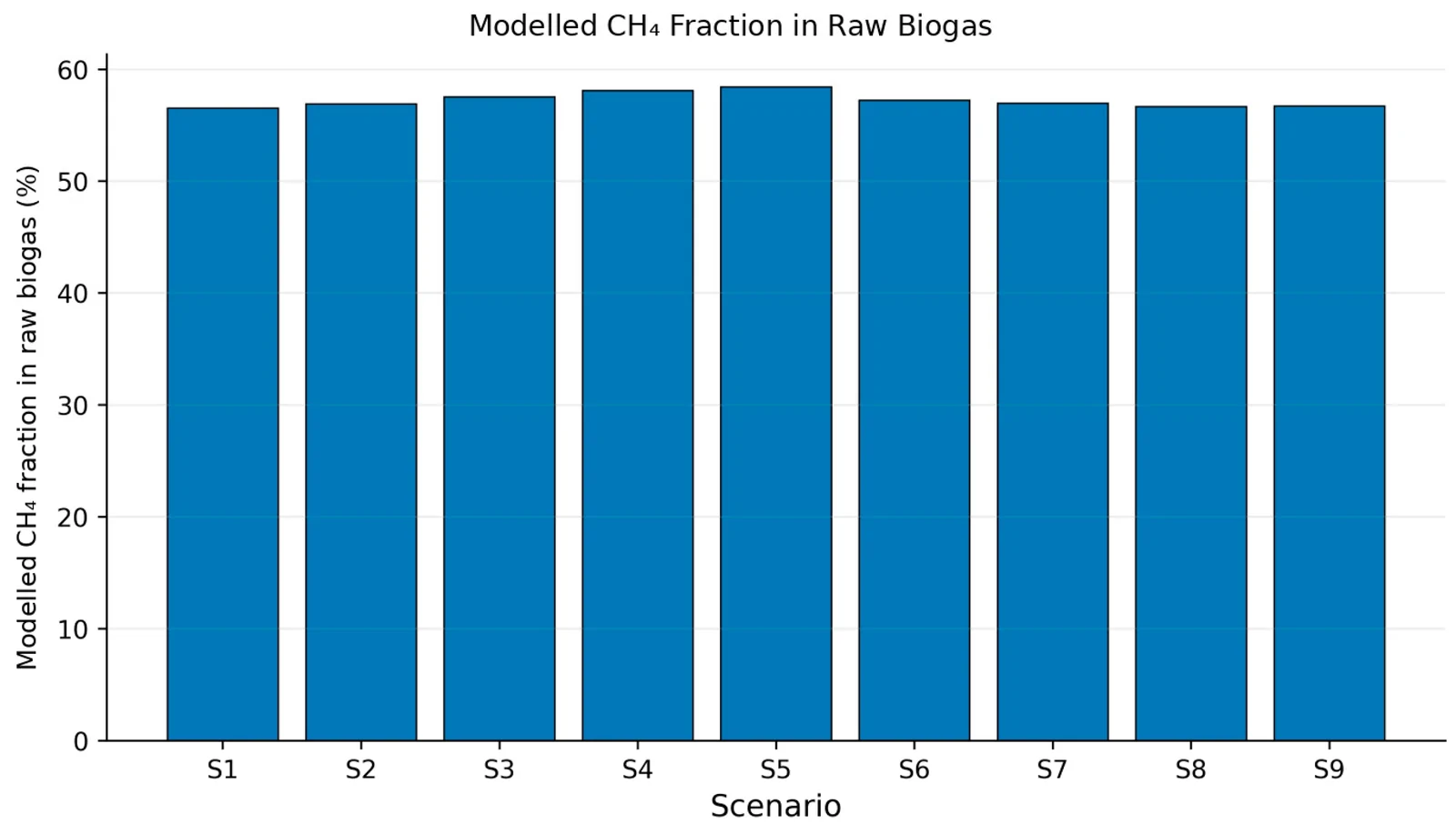
Modelled CH_4_ fraction in raw biogas across S1–S9. The heuristic expression is constrained to 50–70% CH_4_; the values represent raw-biogas composition and not upgraded methane purity.

The screening model generated a raw-biogas CH_4_ fraction of 56.5% for S1 and 58.4% for S5 (Figure 5). The SCG and high-OLR cases remained within the imposed model bound of 50–70% CH_4_. These values are heuristic raw-biogas composition outputs and should not be interpreted as measurements of upgraded methane purity.

#### 2.2.5. Gross Methane Energy Recovery

**Figure 6:**
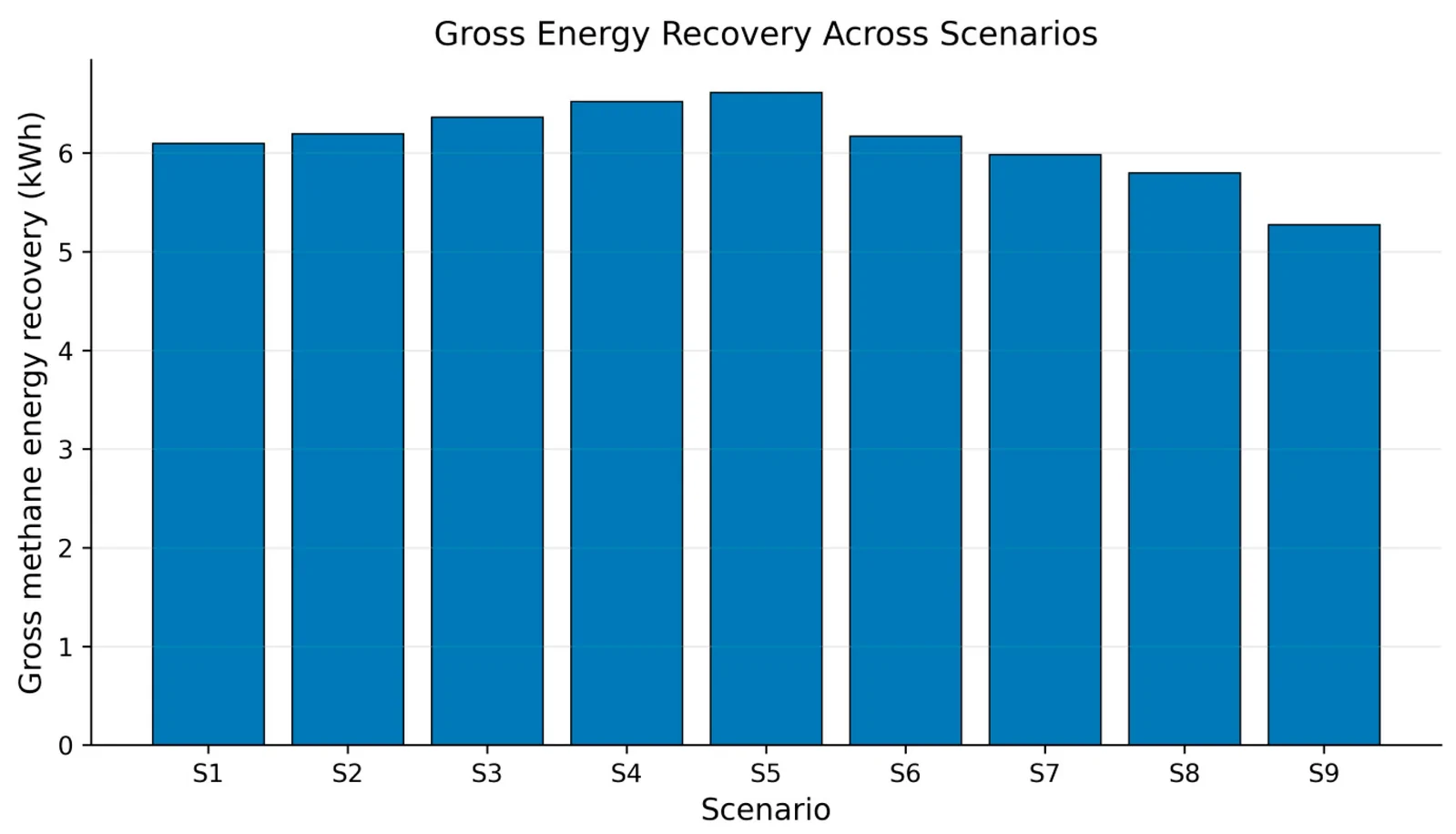
Gross methane energy recovery across S1–S9 on the selected 10 kg wet-mass/2.25 kg VS simulation basis. The values follow modelled methane volume and do not represent net process energy because pretreatment, aeration, mixing, heating, fungal/enzyme production and upgrading energy demands were not subtracted.

Using the 10 kg wet-substrate basis (2.25 kg VS), gross methane energy increased from 6.1 kWh for the control to 6.6 kWh under extended pretreatment (Figure 6). Energy recovery decreased as SCG inclusion increased and was lowest in the high-OLR scenario (5.3 kWh). These values represent gross methane energy only; pretreatment, aeration, mixing, heating, fungal/enzyme production and gas-upgrading energy demands were not subtracted.

#### 2.2.6. Stability and OLR-Related Risk

**Figure 7:**
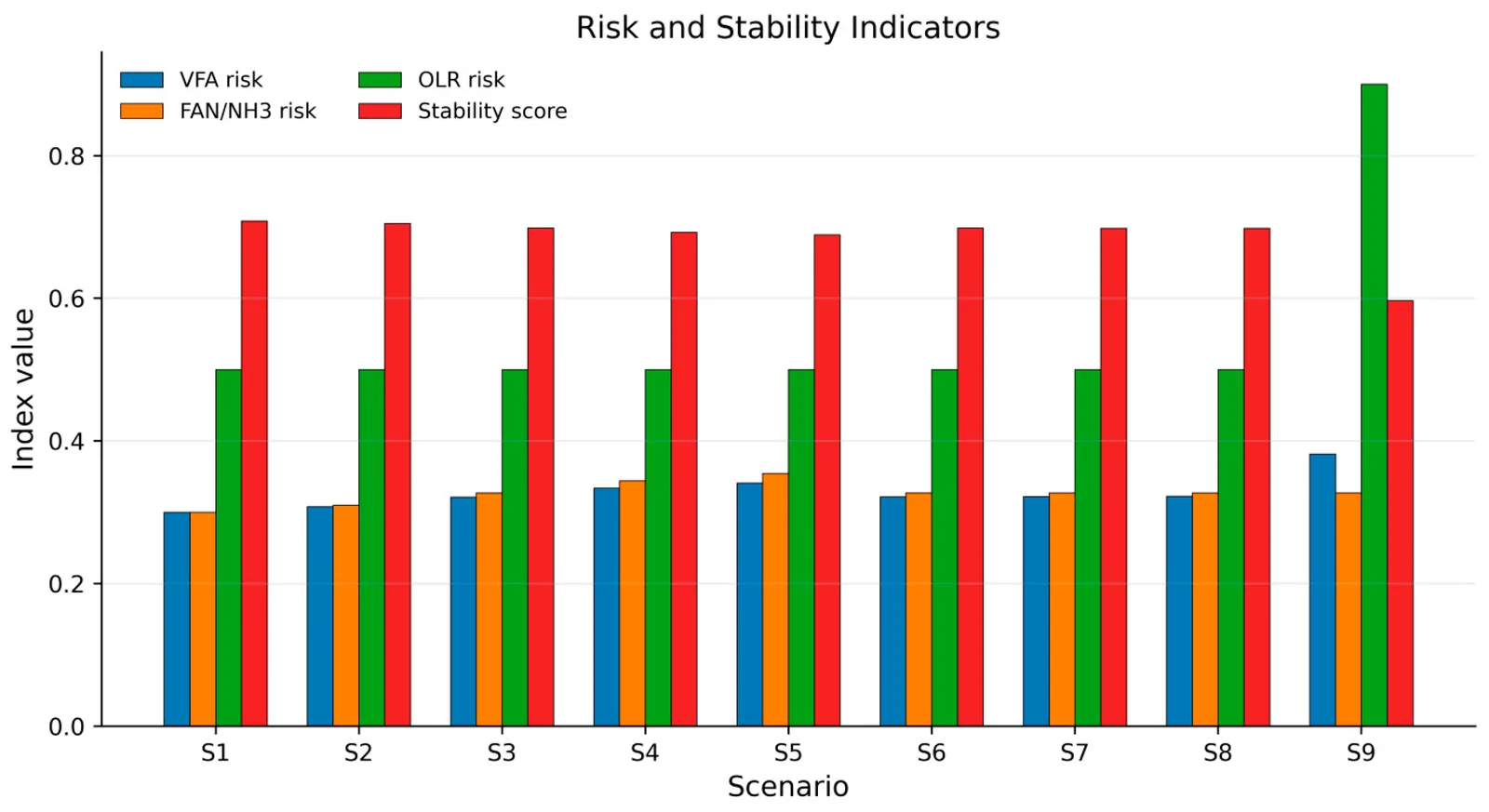
Modelled risk and stability indicators across S1–S9. VFA-, nitrogen- and OLR-related terms and the overall stability score are heuristic screening indices generated by the current equations; they are not validated reactor-stability measurements. The high-OLR stress case shows the strongest modelled OLR penalty.

**Figure 8:**
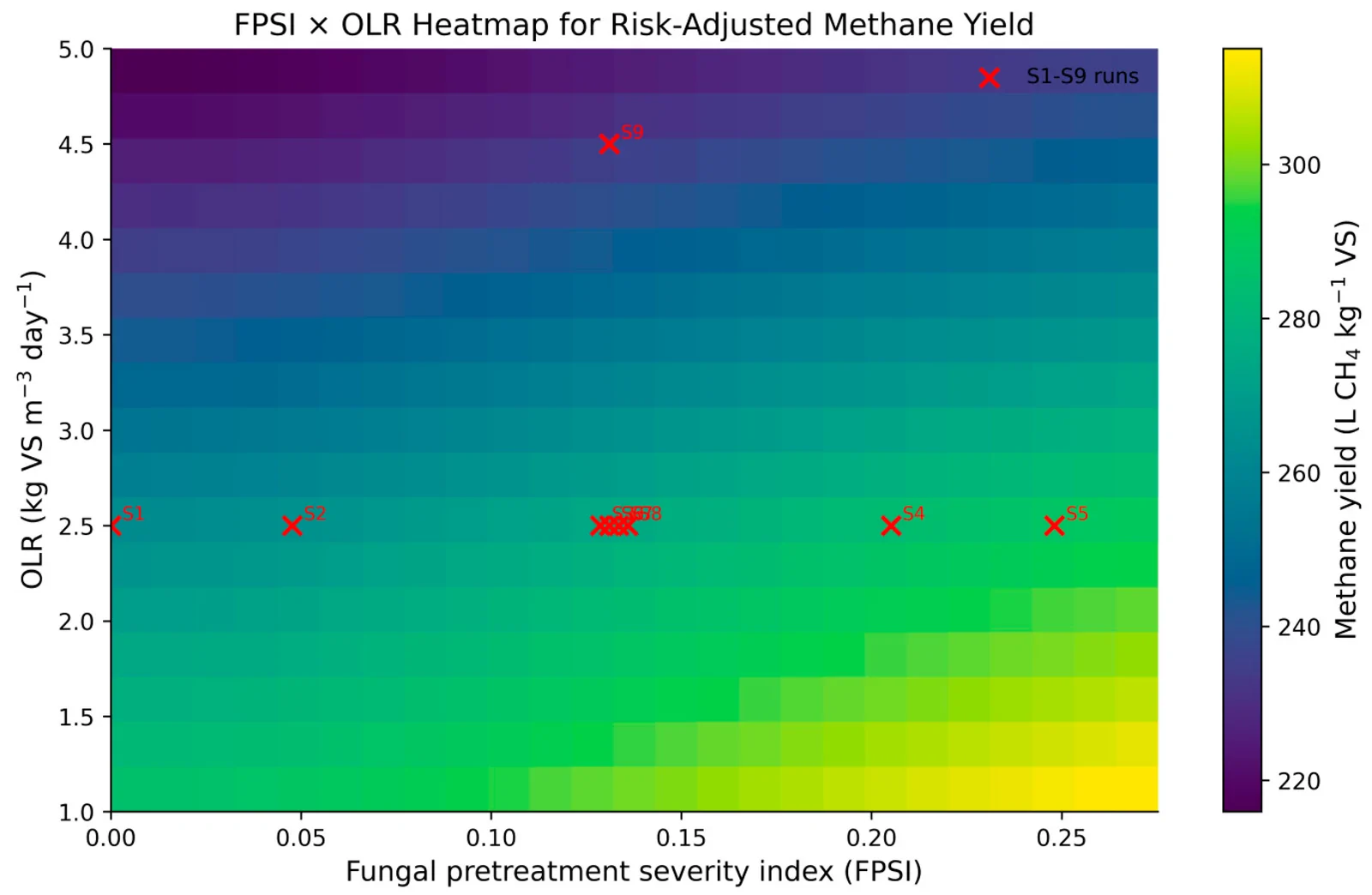
Model-derived FPSI–OLR screening response surface for stability-adjusted methane output. The surface visualises the behaviour generated by the current fungal pretreatment severity index (FPSI) and organic loading rate (OLR) relationships; red markers indicate the final S1–S9 scenarios. The revised surface spans the full FPSI range represented by S1–S9, including the high and extended pretreatment cases. It should not be interpreted as an experimentally validated optimisation map.

The heuristic stability indicator changed from 0.71 in S1 to 0.69 in S5, while the high-OLR stress scenario (S9) generated a lower value of 0.60 (Figure 7). No formal Monte Carlo, replicate-based or probability-distribution uncertainty analysis was performed; therefore, the small deterministic difference between S1 and S5 is not interpreted as statistically or experimentally meaningful. Within the current framework, OLR does not directly reduce biochemical methane potential; instead, OLR enters the weighted risk term and consequently lowers stability-adjusted methane output. Figure 8 therefore visualises the behaviour imposed by the combined FPSI and OLR relationships. It is a scenario-screening surface, not an experimentally validated optimisation map.

#### 2.2.7. Sensitivity Ranking

**Figure 9:**
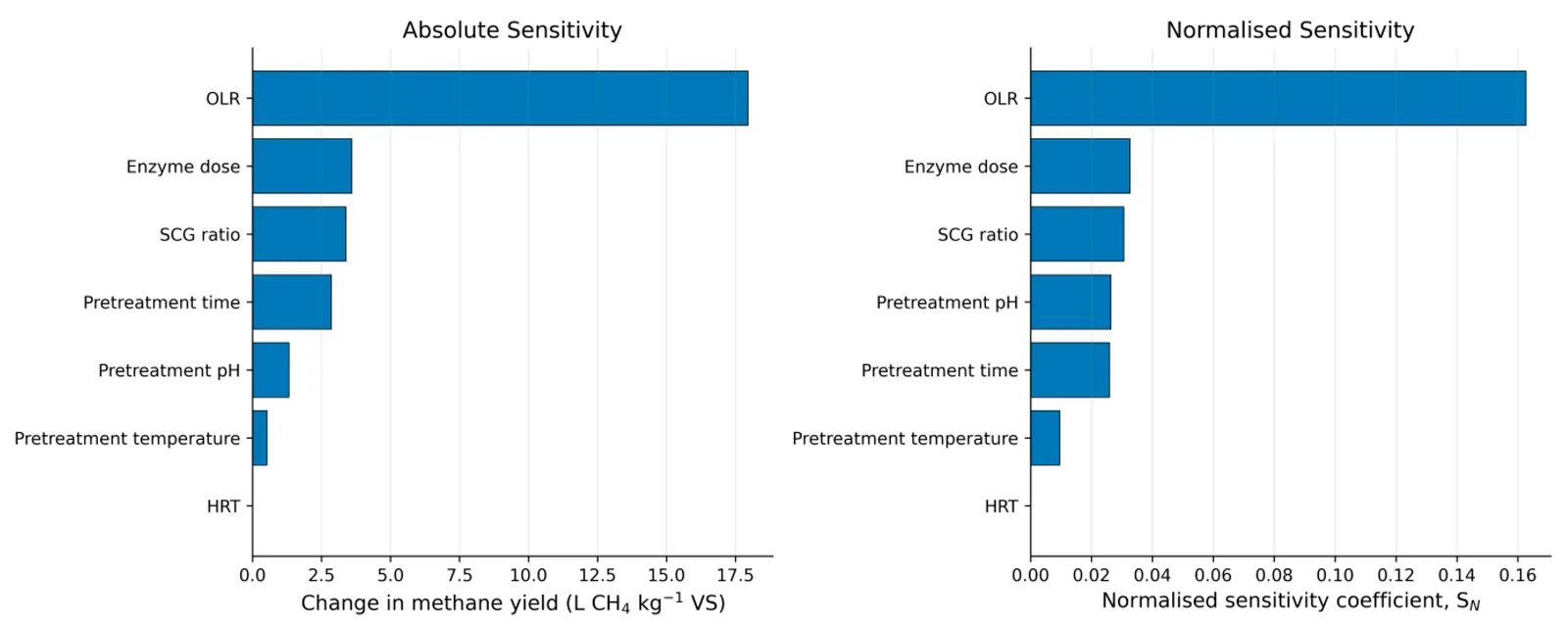
Local sensitivity ranking for the literature-informed screening model. The left panel shows absolute change in stability-adjusted methane yield, whereas the right panel shows the normalised sensitivity coefficient, S_N, allowing comparison across variables tested over different perturbation windows. HRT remains at zero because it is not mechanistically linked to the current model equations and is therefore not interpreted as a biological sensitivity result.

The local one-at-a-time sensitivity analysis identified OLR as the strongest driver of stability-adjusted methane yield within the tested windows (Figure 9). The revised analysis now reports both the absolute methane-yield change and the normalised sensitivity coefficient so that variables tested over different perturbation windows can be compared more fairly. After normalisation, OLR remained the dominant model driver, followed by enzyme dose and SCG ratio; pretreatment pH and pretreatment time produced smaller and broadly similar responses, while pretreatment temperature was lower. HRT produced a zero response because it is retained as a scenario descriptor but is not mechanistically connected to the current methane or stability equations. This zero value is therefore a structural property of the present code and is not evidence that HRT is biologically unimportant.

#### 2.2.8. Candidate Scenarios for Future Laboratory Testing

The extended pretreatment scenario generated the highest modelled methane output, but the current framework does not represent fungal carbon consumption, fungal biomass formation, contamination dynamics or pretreatment energy demand. Extended pretreatment is therefore treated only as an upper-bound screening case and not as an optimum. The high-pretreatment condition is retained as a pragmatic candidate for initial laboratory testing. A future experimental design should compare the untreated control, moderate and high fungal pretreatment, a low-to-moderate SCG condition, and a loading stress case using independently controlled operating variables.

## 3. Discussion

### 3.1. Fungal Pretreatment as a Biochemical Upgrading Layer

A principal behaviour of the framework is that increasing fungal pretreatment severity generates higher modelled methane recovery when FPSI is propagated through solubilisation, hydrolysis and biodegradability terms. This behaviour is partly imposed by the equations and should not be treated as an independent validation of fungal pretreatment efficacy. Its value is instead that the direction of the model response can be compared qualitatively with external experimental literature. Enzyme-rich fungal mash pretreatment has been reported to increase glucose, free amino nitrogen and SCOD together with methane yield and production rate from mixed food waste [32], and broader fungal-pretreatment literature supports improved accessibility of structurally complex biomass [16,20]. The present framework therefore uses these biological observations as plausibility checks, not as independent validation of the same assumptions used to construct the model.

### 3.2. Pretreatment Severity, Methane Yield, and Kinetics

Within the specified model relationships, stability-adjusted methane output increases from 272.5 L CH_4_ kg^−1^ VS in S1 to 291.5–295.5 L CH_4_ kg^−1^ VS in the high and extended pretreatment scenarios. The direction is consistent with the conceptual expectation that improved accessibility and hydrolysis can increase recoverable methane, but it is not independent of the model assumptions because FPSI explicitly modifies solubilisation, biodegradability, hydrolysis and lag terms. Likewise, the modified Gompertz curves show shorter lag and faster accumulation as FPSI increases because the lag-phase modifier is directly linked to FPSI. These outputs are therefore model-imposed scenario behaviours and experimentally testable hypotheses rather than independently validated kinetic findings. Extended treatment must also be considered critically because fungal substrate consumption, biomass formation, longer processing time, contamination risk and energy demand are not represented and may offset theoretical gains.

### 3.3. SCG Trade-Offs and Co-Digestion Interpretation

Within the specified parameterisation, stability-adjusted methane output decreases as SCG increases from 10% to 30% VS. This direction is not an independent validation of a negative SCG effect: it arises from the simplified SCG composition, lower assigned SCG biodegradability and downstream risk relationships used in the framework. The direction is nevertheless qualitatively compatible with continuous co-digestion evidence showing that small SCG additions can exploit spare digestion capacity while progressively higher inclusion may produce diminishing returns or instability [19]. S6–S8 should therefore be interpreted as controlled comparative screening scenarios, not as experimentally established dose–response behaviour or optimum inclusion levels.

### 3.4. OLR, Stability, and Stability-Adjusted Methane Screening Output

OLR was the strongest local and normalised sensitivity driver within the current formula set, and the high-OLR scenario generated the lowest stability-adjusted methane output. This dominance is partly structural because OLR enters the heuristic VFA/risk formulation directly; it should not be interpreted as evidence that OLR is universally more influential than biological pretreatment variables. The current framework does not mechanistically derive OLR from reactor volume and feed rate, nor does it couple OLR to HRT. HRT is retained only as a scenario descriptor and has no equation-level effect. The stability-adjusted formulation is therefore useful for screening model behaviour but is not a substitute for measured VFA, alkalinity, TAN/free-ammonia, pH, feed rate, working volume and HRT profiles.

### 3.5. Translational Significance for Fungal Biotechnology

The paper is intentionally positioned at the interface between fungal biotechnology and process engineering. The framework adds value beyond a narrative literature synthesis by making assumptions explicit and executable, enforcing internally consistent mass and scenario calculations, propagating feedstock and pretreatment assumptions into common downstream outputs, enabling matched scenario and sensitivity comparisons, and generating a reproducible set of candidate conditions and measurements for subsequent experimental testing. The fungal contribution is represented through substrate-targeted enzyme classes and operating variables rather than through a generic pretreatment label. This provides a one-directional computational route from fungal enzyme-related assumptions to process-level screening consequences such as solubilisation, hydrolysis, methane kinetics and heuristic stability risk. The approach also addresses calls in AD modelling for better substrate characterisation and more explicit representation of modified biochemical pathways [5,9,11].

### 3.6. Sustainability, Techno-Economic Considerations, and Future Validation

The current energy outputs are gross methane-energy values, not net-energy or techno-economic results. Future analysis should incorporate fungal culture or enzyme production, fungal biomass formation and substrate consumption, pretreatment time, aeration, mixing, heating, contamination control, gas upgrading and digestate management. This distinction is consistent with process-simulation literature showing that methane yield alone is insufficient for evaluating overall process performance [6]. Similarly, future experimental validation should measure food-waste and SCG composition, enzyme activity, SCOD and soluble-sugar release, free amino nitrogen, TAN/free ammonia, VFA, alkalinity, cumulative methane and raw-biogas CH_4_ fraction. Such measurements would allow the literature-informed framework to progress from scenario screening toward calibrated decision support [5,9,37].

The framework is literature-informed and uses heuristic severity and stability layers. It is not a calibrated fungal-population model, full ADM1 implementation or experimentally validated reactor model. It does not represent fungal substrate consumption or biomass growth, carbonate/alkalinity buffering, mechanistic pH control, or a coupled relationship among feed rate, reactor volume, OLR and HRT. Several model coefficients, allocation factors and critical-risk limits are literature-bounded or internally selected priors and should be treated as uncertainty sources. The CH_4_-fraction equation, risk weighting and objective score are screening constructs for comparative scenario analysis rather than universal process correlations.

Future validation should replace literature-derived and internally selected assumptions with experimentally measured food-waste and SCG composition, enzyme-specific activity, SCOD, soluble sugars, free amino nitrogen, TAN/free ammonia, VFAs, alkalinity, pH, BMP/CSTR methane-production profiles and gas composition. Replicate measurements and independent calibration/validation datasets will be required to quantify predictive error. Global uncertainty analysis, Monte Carlo sampling and factorial designs should then be applied once empirically defensible parameter distributions and independent factor ranges are available. A subsequent techno-economic analysis (TEA) should integrate pretreatment costs, fungal or enzyme production, aeration, mixing, heating, reactor sizing, gas upgrading, CAPEX, OPEX, energy recovery and potential revenues to determine whether candidate screening conditions remain economically viable at larger scale. These activities were intentionally outside the scope of the present study.

## 4. Materials and Methods

### 4.1. Study Design and Scope

This study was designed as a literature-informed computational modelling investigation. Published experimental and modelling studies were used to define the main input ranges for food-waste composition, SCG, fungal pretreatment, AD operation, methane potential, gas composition, energy recovery and process-stability indicators [5,7,8,9,10,11,12,13,14,15,16,17,18,19,28,29,31,32,33,36,37]. No new laboratory measurements were generated. The purpose of the model was to convert fungal-biotechnology information into a reproducible simulation framework linking enzyme-related pretreatment with substrate solubilisation, hydrolysis, methane production, raw-biogas CH_4_ fraction, energy recovery and operational risk.

The final simulation was implemented in Python (version 3.13.15) using Google Colaboratory (Google LLC, Mountain View, CA, USA). All simulations and data analyses were performed using NumPy (version 2.1.3), pandas (version 2.2.3), and Matplotlib (version 3.10.0). One final S1–S9 Python run was used as the master dataset so that all result tables and figures are internally consistent. The exact final simulation notebook/script, generated output files, and figures from the final model run are archived in Zenodo [35] (https://doi.org/10.5281/zenodo.22827017) The development repository is also available at https://github.com/Mursalin1075/fungal-biomethane-simulation-framework (accessed on 4 October 2026).

### 4.2. Model Architecture

The model follows the sequential pathway: feed composition -> SCG mixing -> fungal pretreatment -> substrate solubilisation -> hydrolysis and lag-phase modification -> methane production -> raw-biogas composition -> gross energy recovery -> heuristic stability/risk scoring -> scenario comparison. Inputs were grouped as feedstock (wet mass, TS, VS and VS/TS), composition (carbohydrate, protein, lipid and inert fractions), fungal pretreatment (enzyme activity, time, temperature and pH), AD operation (OLR, HRT, initial pH and mesophilic temperature) and stability inputs (baseline SCOD, soluble sugar, free amino nitrogen, TAN, VFA and critical limits). HRT is retained only as a scenario descriptor in the current implementation and does not enter the methane or stability equations. OLR is used as an independent screening input and is not mechanistically calculated from feed rate and reactor working volume or coupled to HRT.

The model reports both an unpenalised biochemical methane output and a stability-adjusted methane screening output. The latter is a heuristic comparative metric obtained by multiplying the biochemical methane term by the uncalibrated stability factor. This distinction separates substrate-derived methane potential from the internally imposed adjustment associated with the screening stability terms. Accordingly, the quantitative outputs should be interpreted as deterministic scenario-screening results that generate hypotheses for future experimental testing, rather than as experimentally calibrated reactor predictions or expected process performance.

### 4.3. Literature-Derived Parameter Selection and Mass Basis

The model parameters were treated as literature-informed priors rather than experimentally calibrated values. The current simulation uses a 10 kg wet-substrate basis, with TS = 0.25 and VS/TS = 0.90, giving 2.5 kg TS and 2.25 kg VS. Methane yield is normalised to L CH_4_ kg^−1^ VS, while total energy outputs reported in kWh refer to the 2.25 kg VS basis. The wet-mass, total-solids, volatile-solids and compositional mass-balance relationships are defined in Equations (1)–(3).

*M*_TS_ = *M*_wet_ × *f*_TS_ (1)

*M*_VS_ = *M*_TS_ × *f*_VS/TS_ (2)

*f*_carb_ + *f*_protein_ + *f*_lipid_ + *f*_inert_ = 1 (3)

The baseline food-waste fractions were carbohydrate = 0.50, protein = 0.25, lipid = 0.20 and inert = 0.05, with baseline biodegradability = 0.72. SCG fractions were carbohydrates = 0.42, protein = 0.16, lipid = 0.22 and inert = 0.20, with baseline biodegradability = 0.55. The theoretical methane-potential coefficients were 0.415, 0.496 and 1.014 Nm^3^ CH_4_ kg^−1^ VS for carbohydrate, protein and lipid fractions, respectively [7,8]. SCG composition and co-digestion behaviour were bounded using published characterisation and digestion studies [14,15,16,17,18,19]. The principal literature-informed parameter ranges used to define the simulation space are summarised in Table 3. Parameter bounds were selected from literature-reported values where directly available and were otherwise defined as literature-informed screening intervals for comparative scenario analysis. These ranges should not be interpreted as universal experimentally validated operating limits.

**Table 3:** Principal literature-informed parameter ranges and model screening bounds used to define the simulation space.

Parameter	Literature-Informed Range/ Screening Bound	References
Food waste TS	15–35% wet basis	[2,10]
VS/TS	80–95%	[2,10]
Carbohydrate fraction	40–70% VS	[2,10]
Protein fraction	10–30% VS	[2,10]
Lipid fraction	5–30% VS	[2,10,13]
Mixed food-waste BMP	0.35–0.65 Nm^3^ CH_4_ kg^−1^ VS	[2,8,29]
SCG screening ratio	0–30% VS	[14,19]
Pretreatment time	12–72 h	[32,34]
Pretreatment temperature	25–35 °C	[34]
Pretreatment pH	4.5–6.5	[34]
AD temperature	35 ± 2 °C	[28,29]
AD pH	6.8–7.5	[28,29]
OLR	1–4 kg VS m^−3^ d^−1^	[28,29,30]
HRT	15–30 d	[28,29]

### 4.4. Fungal Pretreatment Representation

Fungal pretreatment was represented using a fungal pretreatment severity index (FPSI). FPSI is a heuristic modelling layer rather than a full fungal-population model. It translates enzyme-related pretreatment conditions into a bounded severity term that modifies solubilisation, hydrolysis, lag phase and biodegradability. The approach is biologically motivated by fungal/enzymatic pretreatment studies, but the response functions, allocation factors and weighting coefficients are part of the present modelling framework and have not been calibrated against enzyme-specific experimental kinetics [16,20,32,33]. The fixed values used in the final simulation, together with their modelling role and assumption status, are reported in Table 1. The enzyme-activity and FPSI response functions are defined in Equations (4)–(9), with pretreatment-linked solubilisation, hydrolysis and lag modifiers given in Equations (10)–(15).

*A_i_* = *D_i_* × *T_i_* × *P_i_* × *R_i_* (4)

(5) $$D_{i}=\frac{E_{i}}{K_{E,i}+E_{i}}$$

(6) $$T_{i}=\exp\left[-{\left(\frac{T_{\mathrm{pre}}-T_{\mathrm{opt},i}}{\sigma_{T,i}}\right)}^{2}\right]$$

(7) $$P_{i}=\exp\left[-{\left(\frac{{\mathrm{pH}}_{\mathrm{pre}}-{\mathrm{pH}}_{\mathrm{opt},i}}{\sigma_{\mathrm{pH},i}}\right)}^{2}\right]$$

*R_i_* = 1 − exp(−*k_t_*_,*i*_*t*_pre_) (8)

(9) $$\mathrm{FPSI}=\sum_{i}w_{i}A_{i}$$

FPSI was constrained to 0–1. Weighting factors were 0.35 for carbohydrate-targeting effects, 0.25 for protein-targeting effects, 0.20 for lipid-targeting effects and 0.20 for SCG-supporting effects. Because class-specific measured enzyme activities were unavailable, total enzyme activity was distributed using internal allocation factors of 0.40, 0.30, 0.20 and 0.10, respectively. These weights and allocation factors are heuristic assumptions, not fitted biological constants. Alternative weight combinations were not independently calibrated in the present study; therefore, their influence is treated as an uncertainty source and should be assessed and recalibrated when enzyme-specific measurements become available.

SCOD_pretreated_ = SCOD_control_(1 + *α*_SCOD_ FPSI) (10)

Sugar_pretreated_ = Sugar_control_(1 + *α*_sugar_*A*_carb_) (11)

FAN_release_ = FAN_control_(1 + *α*_FAN_*A*_protein_) (12)

TAN_pretreated_ = TAN_control_(1 + *α*_TAN_*A*_protein_) (13)

Here, FAN denotes free amino nitrogen released during protein hydrolysis. It is distinct from TAN and free ammonia (NH3-N), which are relevant to AD inhibition. Full free-ammonia speciation is not included in the current model.

*k*_hyd,modified_ = *k*_hyd,base_(1 + *α*_hyd_ FPSI) (14)

*λ*_modified_ = *λ*_base_(1 − *α_λ_* FPSI) (15)

### 4.5. Methane, Raw-Biogas, Energy, and Stability Calculations

Substrate mixing and methane calculations are defined in Equations (16)–(23), raw-biogas composition in Equations (24)–(26), gross energy calculations in Equations (27)–(29), and heuristic risk/stability/objective calculations in Equations (30)–(39).

*f_i_*_,mix_ = (1 − *f*_SCG_)*f_i_*_,FW_ + *f*_SCG_*f_i_*_,SCG_ (16)

*M_i_* = *M*_VS_ × *f_i_*_,mix_ (17)

(18) $${\mathrm{BMP}}_{\mathrm{mix}}=\sum_{i}f_{i}\;{\mathrm{BMP}}_{i}\;$$

*B_i_*_,treated_ = *B_i_*_,control_ + (1 − *B_i_*_,control_)*A_i_* (19)

(20) $$V_{{CH}_{4},i}=M_{i}\times {\mathrm{BMP}}_{i}\times B_{i}$$

(21) $$V_{{CH}_{4},\mathrm{biochemical}}=\sum_{i}V_{CH_{4},i}$$

(22) $$V_{{CH}_{4},\mathrm{practical}}=V_{{CH}_{4},\mathrm{biochemical}}\times \mathrm{Stability}$$

(23) $$Y_{{CH}_{4}}=\frac{V_{{CH}_{4},\mathrm{practical}}}{M_{\mathrm{VS}}}$$

The high-OLR scenario is reduced through the stability penalty rather than by directly lowering biochemical methane potential. The modelled CH_4_ fraction in raw biogas was estimated using the following heuristic expression and constrained between 0.50 and 0.70, a broad range consistent with raw food-waste biogas literature [2,8,29]:

(24) $$f_{{CH}_{4}}=0.58+0.08\;\mathrm{FPSI}-0.05R_{\mathrm{total}}-0.03f_{\mathrm{SCG}}$$

(25) $$V_{\mathrm{biogas}}=\frac{V_{{CH}_{4},\mathrm{practical}}}{f_{{CH}_{4}}}$$

(26) $$V_{CO_{2}}=V_{\mathrm{biogas}}-V_{{CH}_{4},\mathrm{practical}}$$

Gross methane energy recovery was calculated from the lower heating value of methane:

(27) $$E_{{CH}_{4}}\;\left(\mathrm{MJ}\right)=V_{{CH}_{4},\mathrm{practical}}\;\left(m^{3}\right)\times 35.8$$

(28) $$E_{{CH}_{4}}\;\left(\mathrm{kWh}\right)=\frac{E_{{CH}_{4}}\;\left(\mathrm{MJ}\right)}{3.6}$$

(29) $$E_{\mathrm{electricity}}=E_{{CH}_{4}}\times 0.35$$

The 0.35 factor is an assumed CHP electrical efficiency. Net energy was not calculated because pretreatment energy demand, aeration, mixing, heating, enzyme preparation and upgrading costs were outside the current scope.

Stability was represented by VFA, nitrogen, pH and OLR risk terms. These are heuristic screening indicators informed by established AD inhibition behaviour [12,13,31], not validated reactor-stability equations. The VFA–FPSI coefficient (0.55), VFA–OLR coefficient (0.25), pH-response coefficient (0.45), critical limits and risk weights were selected as literature-informed/internal screening priors and are listed explicitly in Table 1. They should be recalibrated against measured VFA, alkalinity, TAN/free-ammonia and pH data before quantitative reactor prediction is attempted. The pH expression is an empirical screening relationship and does not represent carbonate alkalinity, bicarbonate speciation or buffering capacity. Similarly, OLR is not mechanistically coupled to reactor working volume, feed rate or HRT in the current implementation.

(30) $${\mathrm{VFA}}_{\mathrm{est}}={\mathrm{VFA}}_{\mathrm{control}}\left[1+0.55\;\mathrm{FPSI}+0.25\max\left(\frac{\mathrm{OLR}-{\mathrm{OLR}}_{\mathrm{base}}}{{\mathrm{OLR}}_{\mathrm{base}}},0\right)\right]$$

(31) $${\mathrm{pH}}_{\mathrm{actual}}={\mathrm{pH}}_{\mathrm{initial}}-0.45\left(\frac{{\mathrm{VFA}}_{\mathrm{est}}}{{\mathrm{VFA}}_{\mathrm{crit}}}\right)$$

(32) $$R_{\mathrm{VFA}}=\frac{{\mathrm{VFA}}_{\mathrm{est}}}{{\mathrm{VFA}}_{\mathrm{crit}}}$$

(33) $$R_{\mathrm{FAN}}=\frac{{\mathrm{FAN}}_{\mathrm{release}}}{{\mathrm{FAN}}_{\mathrm{crit}}};\;\;R_{\mathrm{TAN}}=\frac{{\mathrm{TAN}}_{\mathrm{pretreated}}}{{\mathrm{TAN}}_{\mathrm{crit}}}$$

*R_N_* = max(*R*_FAN_,*R*_TAN_) (34)

(35) $$R_{\mathrm{pH}}=\frac{\left|{\mathrm{pH}}_{\mathrm{actual}}-{\mathrm{pH}}_{\mathrm{opt}}\right|}{{\mathrm{pH}}_{\mathrm{opt}}};\;\;R_{\mathrm{OLR}}=\frac{\mathrm{OLR}}{{\mathrm{OLR}}_{\mathrm{crit}}}$$

*R*_total_ = 0.35*R*_VFA_ + 0.25*R_N_* + 0.20*R*_pH_ + 0.20*R*_OLR_ (36)

Stability = 1 − *R*_total_ (37)

Stability was constrained between 0 and 1. The overall objective score was used only for comparative scenario ranking:

Objective = *Y*_norm_ + *P*_norm_ + *E*_norm_ − *λR*_total_ (38)

(39) $$Y_{\mathrm{norm}}=\frac{Y_{{CH}_{4}}}{650};\;\;P_{\mathrm{norm}}=\frac{P_{{CH}_{4}}}{100};\;\;E_{\mathrm{norm}}=\frac{E_{{CH}_{4},\mathrm{kWh}}}{10};\;\;\lambda =0.80$$

### 4.6. Scenario Design

Nine scenarios were defined as shown in Table 2. S1 was untreated control. S2–S4 intentionally co-varied enzyme dose and pretreatment duration to represent increasing combined pretreatment severity and therefore cannot be used to infer independent dose and time effects. S5 retained the high enzyme dose (90 U g^−1^ VS) while extending pretreatment from 36 to 72 h, providing a time-focused upper-bound comparison. S6-S8 held moderate pretreatment (60 U g^−1^ VS, 24 h) and OLR (2.5 kg VS m^−3^ d^−1^) constant while SCG increased from 10% to 30%, providing the clearest matched SCG comparison. S9 retained moderate pretreatment and 10% SCG while increasing OLR from 2.5 to 4.5 kg VS m^−3^ d^−1^ and changing the HRT descriptor from 25 to 20 d. Because HRT has no equation-level effect, the modelled S6–S9 difference arises from the OLR risk term rather than from HRT. The scenario set is not a full factorial design, and independent main and interaction effects will require a future factorial study. Extended pretreatment and SCG levels are screening/stress conditions rather than established optima [19].

Methane-improvement percentages were calculated within each scenario relative to that scenario’s own untreated biochemical methane output, using $\mathrm{Improvement}\;\left(\%\right)=\frac{{CH_{4}}_{\mathrm{treated}}-{CH_{4}}_{\mathrm{control}}}{{CH_{4}}_{\mathrm{control}}}\times 100$. For S2–S5, the comparator is untreated food waste. For S6–S9, the comparator is the matched untreated mixed-substrate condition with the same SCG composition (and corresponding scenario basis), rather than the S1 food-waste-only control. Accordingly, the SCG bars represent the pretreatment-associated improvement within each substrate composition and should not be interpreted as percentage improvement relative to S1.

### 4.7. Modified Gompertz Representation

Cumulative methane curves were generated using the modified Gompertz formulation, a widely used empirical representation of microbial gas-production kinetics [36], using Equations (40) and (41):

(40) $$M\left(t\right)=P\exp\left\{-\exp\left[\left(\frac{R_{m}e}{P}\right)\left(\lambda -t\right)+1\right]\right\}$$

(41) $$P=V_{{CH}_{4},\mathrm{practical}};\;\;R_{m}=P\left(0.07+0.10\;\mathrm{FPSI}\right);\;\;\lambda =\lambda_{\mathrm{modified}}$$

The curves are illustrative model-generated cumulative methane trajectories. They were not fitted to experimental BMP data and are used only for scenario comparison rather than kinetic validation.

### 4.8. Sensitivity Analysis and Literature-Based Model Evaluation

A one-at-a-time sensitivity analysis was applied around the moderate + 10% SCG reference case (SCG = 0.10, enzyme dose = 60 U g^−1^ VS, pretreatment temperature = 30 °C, pretreatment pH = 5.5, pretreatment time = 24 h, OLR = 2.5 kg VS m^−3^ d^−1^, HRT = 25 d). The exact low/high values were: SCG ratio 0.08/0.12; enzyme dose 48/72 U g^−1^ VS; pretreatment temperature 27/33 °C; pretreatment pH 5.0/6.0; pretreatment time 19.2/28.8 h; OLR 2.0/3.0 kg VS m^−3^ d^−1^; and HRT 20/30 d. Absolute sensitivity was calculated as the magnitude of the yield difference between the high and low cases using Equation (42):

Δ*Y* = |*Y*_high_ − *Y*_low_| (42)

A normalised coefficient was additionally calculated using Equation (43) for more equitable comparison across parameters with different perturbation windows:

(43) $$S_{X}=\frac{\left(Y_{\mathrm{high}}-Y_{\mathrm{low}}\right)/Y_{\mathrm{base}}}{\left(X_{\mathrm{high}}-X_{\mathrm{low}}\right)/X_{\mathrm{base}}}$$

Because HRT is accepted by the scenario function but is not used by the current methane or stability equations, its calculated sensitivity is exactly zero. HRT is therefore retained only as a scenario descriptor in the present version and its zero response is not interpreted as a biological conclusion. A global sensitivity or Monte Carlo analysis was not performed because experimentally calibrated probability distributions were unavailable for several heuristic coefficients. Consequently, the manuscript does not report confidence intervals and the deterministic sensitivity results should be interpreted only as local model-behaviour screening.

The model was evaluated using literature-based consistency checks rather than new experimental validation. Modelled methane output, raw-biogas CH_4_ fraction, SCG response, fungal-pretreatment response and OLR response were compared qualitatively with ranges and directional behaviour reported in published studies [2,5,8,9,19,28,29,31,32,37]. The literature used to define model assumptions is not treated as independent validation of those same assumptions.

## 5. Conclusions

This study presents a literature-informed scenario-screening framework that translates fungal pretreatment descriptors for heterogeneous food waste into model-ready AD variables. By representing fungal effects through substrate solubilisation, hydrolysis, biodegradability, methane kinetics, raw-biogas CH_4_ fraction, gross energy recovery and heuristic risk indicators, the framework provides a one-directional computational translation from fungal-pretreatment assumptions to AD-relevant screening outputs.

Under the specified model assumptions, the selected S1–S9 scenarios generate higher heuristic stability-adjusted methane outputs with increasing combined fungal pretreatment severity, while increasing SCG proportion and high OLR generate lower outputs and/or higher heuristic risk. These directional responses are partly imposed by the model architecture: FPSI explicitly modifies solubilisation, biodegradability, hydrolysis and lag, while SCG composition and OLR enter the substrate and risk calculations. Accordingly, the quantitative outputs are interpreted as deterministic scenario-screening results and experimentally testable hypotheses, rather than expected process performance, independent quantitative predictions or validated operating optima. Independent experimental calibration and validation, uncertainty analysis, factorial testing and techno-economic assessment are required before the framework can support quantitative process design or optimisation.

## Data Availability

No new experimental measurements were generated in this study. The literature-derived parameter ranges, scenario definitions and model-generated outputs supporting the reported results are contained within the article. The exact final simulation notebook/script, generated CSV/output files and figures from the final model run are permanently archived on Zenodo [35] at https://doi.org/10.5281/zenodo.22827017. This Zenodo record constitutes the citable software archive associated with the final model run. The development repository remains available at https://github.com/Mursalin1075/fungal-biomethane-simulation-framework (accessed on 4 October 2026). Software reuse is subject to the licence specified in the archived record.

## Abbreviations

The following abbreviations are used in this manuscript:

AD	Anaerobic digestion
ADM1	Anaerobic Digestion Model No. 1
BMP	Biochemical methane potential
CHP	Combined heat and power
CSTR	Continuously stirred tank reactor
FAN	Free amino nitrogen
FPSI	Fungal pretreatment severity index
HRT	Hydraulic retention time
OLR	Organic loading rate
SCG	Spent coffee grounds
SCOD	Soluble chemical oxygen demand
TAN	Total ammonia nitrogen
TS	Total solids
VFA	Volatile fatty acids
VS	Volatile solids
TEA	Techno-economic analysis
